# 1,1,2,2-Tetra­kis[2,4-dichloro-6-(dieth­oxy­meth­yl)phen­oxy­meth­yl]ethene

**DOI:** 10.1107/S1600536812038299

**Published:** 2012-09-26

**Authors:** Yavuz Köysal, Sema Öztürk Yildirim, Ray J. Butcher, Esra Düğdü

**Affiliations:** a Yeşilyurt Demir Çelik Vocational School, Ondokuz Mayis University, Samsun, Turkey; b Department of Chemistry, Howard University, 525 College Street NW, Washington, DC 20059, USA; c Department of Physics, Faculty of Sciences, Erciyes University, 38039 Kayseri, Turkey; d Department of Chemistry, Karadeniz Technical University, 61080 Trabzon, Turkey

## Abstract

In the title compound, C_50_H_60_Cl_8_O_12_, the mol­ecules are disordered about an inversion center located at the mid-point of the central C=C bond. These atoms show disorder and were modelled with two different orientations with site occupancies of 0.828 (3) and 0.172 (3). The dihedral angle between the two benzene rings in the asymmetric unit is 52.80 (6)°. Intramolecular C—H⋯O and C—H⋯Cl interactions occur and the crystal packing features inversion dimers linked by pairs of C—H⋯O bonds, generating *R*_2_^2^(10) loops.

## Related literature

For anti-oxidant, anti-inflammatory, chemopreventive, anti­bacterial, anti­carcinogenic, anti­tumor and anti­viral properties of sterically hindered phenols and secondary aromatic amines, see: Amorati *et al.* (2003 ▶); Torres de Pinedo *et al.* (2007 ▶); Leopoldini *et al.* (2011 ▶); Leiro *et al.* (2011 ▶); Link *et al.* (2010 ▶); Daglia (2011 ▶); Bai *et al.*, (2003 ▶); Song *et al.* (2005 ▶); Rabek (1990 ▶); Pospisil *et al.* (2003 ▶); Wolf & Kaul (1992 ▶); Thapa *et al.* (2012 ▶). For synthetic phenolic anti­oxidants, such as butyl­ated hy­droxy­toluene (BHT), butyl­ated hy­droxy­anisole (BHA) or butyl­ated hy­droxy­quinone (TBHQ), which possess good anti-oxidant capacity, see: Omura (1995 ▶). For phenols capable of propagation termination due to the donation of the hydrogen atom of the phenolic OH to the free radicals, see: Kumar & Naik (2010 ▶); Findik *et al.* (2011 ▶). For bond lengths of structurally related mol­ecules, see: Öztürk Yildirim *et al.* (2012 ▶). For a description of the Cambridge structural Database, see: Allen (2002 ▶). For details of the synthesis, see: Er *et al.* (2009 ▶).
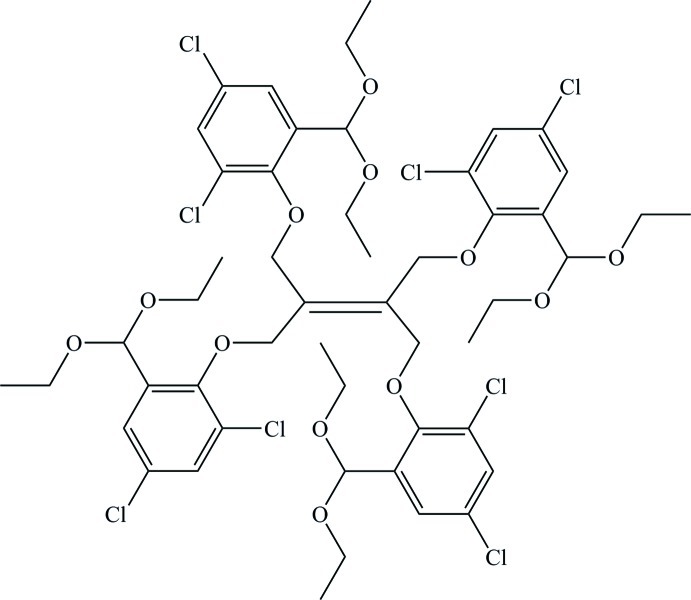


## Experimental

### 

#### Crystal data

C_50_H_60_Cl_8_O_12_
*M* *_r_* = 1136.58
Triclinic, 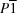

*a* = 8.0626 (3) Å
*b* = 12.8693 (5) Å
*c* = 13.9968 (6) Å
α = 97.425 (3)°
β = 102.878 (3)°
γ = 105.391 (3)°
*V* = 1337.29 (9) Å^3^
*Z* = 1
Mo *K*α radiation
μ = 0.48 mm^−1^
*T* = 123 K
0.62 × 0.19 × 0.07 mm

#### Data collection

Agilent Xcalibur (Ruby, Gemini) diffractometer
Absorption correction: multi-scan [*CrysAlis RED* (Agilent, 2011 ▶), based on expressions derived from Clark & Reid (1995 ▶)] *T*_min_ = 0.755, *T*_max_ = 0.967
10254 measured reflections
6125 independent reflections
5231 reflections with *I* > 2σ(*I*)
*R*_int_ = 0.020

#### Refinement

*R*[*F* ^2^ > 2σ(*F* ^2^)] = 0.038
*wR*(*F* ^2^) = 0.090
*S* = 1.03
6125 reflections
336 parameters
12 restraints
H-atom parameters constrained
Δρ_max_ = 0.45 e Å^−3^
Δρ_min_ = −0.39 e Å^−3^

### 

Data collection: *CrysAlis PRO* (Agilent, 2011 ▶); cell refinement: *CrysAlis PRO*; data reduction: *CrysAlis PRO*; program(s) used to solve structure: *SHELXS97* (Sheldrick, 2008 ▶); program(s) used to refine structure: *SHELXL97* (Sheldrick, 2008 ▶); molecular graphics: *SHELXTL* (Sheldrick, 2008 ▶); software used to prepare material for publication: *SHELXTL*.

## Supplementary Material

Crystal structure: contains datablock(s) I, global. DOI: 10.1107/S1600536812038299/bt6833sup1.cif

Structure factors: contains datablock(s) I. DOI: 10.1107/S1600536812038299/bt6833Isup2.hkl

Supplementary material file. DOI: 10.1107/S1600536812038299/bt6833Isup3.cml

Additional supplementary materials:  crystallographic information; 3D view; checkCIF report

## Figures and Tables

**Table 1:** Hydrogen-bond geometry (Å, °)

*D*—H⋯*A*	*D*—H	H⋯*A*	*D*⋯*A*	*D*—H⋯*A*
C12—H12*B*⋯Cl2	0.99	2.66	3.180 (3)	113
C14—H14*B*⋯O4	0.99	2.50	3.166 (3)	125
C17—H17*A*⋯O2^i^	0.95	2.47	3.3943 (18)	166

Symmetry code: (i) 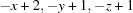.

**Fig. 1.**
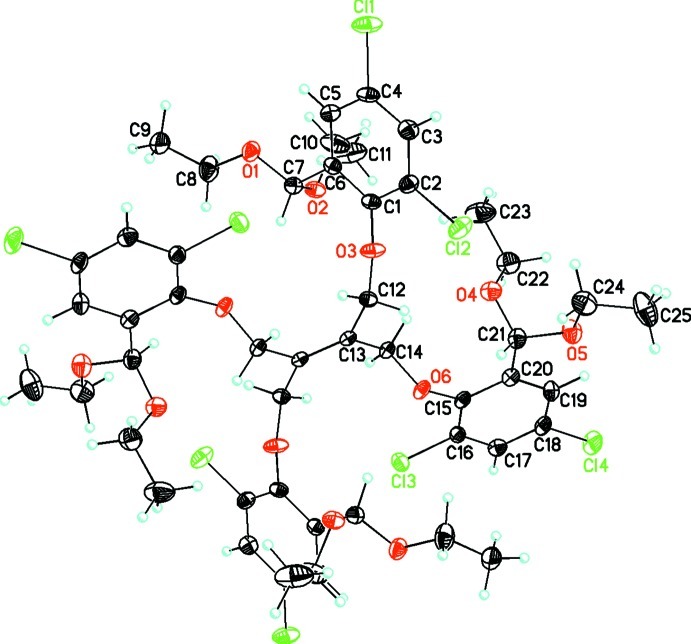
The molecular structure of (I) showing displacement ellipsoids at the 50% probability level [symmetry code: (A) = 1 - x, 1 - y, 1 - z].

## supplementary crystallographic information

### Comment

Many phenolic or polyphenolic compounds have been reported to have a wide range of biological activities such as antioxidant (Amorati *et al.*, 2003; Torres de Pinedo *et al.*, 2007; Leopoldini *et al.*, 2011), anti-inflammatory (Leiro *et al.*, 2011), chemoprevention (Link *et al.*, 2010), antibacterial (Daglia, 2011), anti-carcinogenic and anti-tumor (Bai *et al.*, 2003), and antiviral (Song *et al.*, 2005) activities. The most active antioxidants typically comprise sterically hindered phenols and secondary aromatic amines (Rabek, 1990; Pospisil *et al.*, 2003; Wolf & Kaul, 1992; Thapa *et al.*, 2012). In addition, phenols have been utilized extensively for food preservation. Synthetic phenolic antioxidants, such as butylated hydroxytoluene (BHT), butylated hydroxyanisole (BHA) or butylated hydroxyquinone (TBHQ) possess good antioxidant capacity (Omura, 1995). The main structural feature responsible for the anti-oxidative and free radical scavenging activity of phenolic derivatives is the phenolic hydroxyl group. Phenols are able of donating the hydrogen atom of the phenolic OH to the free radicals, thus stopping the propagation chain during the oxidation process (Kumar & Naik, 2010; Findik, *et al.*, 2011). In view of the importance of such phenolate compounds the structure of 2,2'-((2-(1,3-bis(2,4-dichloro-6-(diethoxymethyl)phenoxy)propan-2-ylidene) propane-1,3-diyl)bis(oxy))bis(1,5-dichloro-3-(diethoxymethyl)benzene) was determined.

The title compound, (Fig. 1), lies on an inversion centre, giving one half-molecule per asymmetric unit which passes through middle point of the C13=C13A double bond of the aliphatic chain. Atoms C12 C13 and C14 atoms show disorder and were modelled with two different orientations and with site occupancies of 0.828 (4):0.172 (4). The (diethoxymethyl)benzene groups adopts an all-*trans* conformation and the molecular structure is not planar. The O3 C12 C13 C14 and C12 C13 C14 O6 torsion angles are 68.6 (2) ° and 82.5 (2) ° and the dihedral angle between the planes of the benzene rings (C1/C6 to C15/C20) is 52.80 (6) ° [for the non-H atoms, maximum deviation = 0.007 (1) Å for C2]. Bond lengths and angles can be regarded as normal for such structures (Öztürk Yildirim *et al.* 2012; Allen, 2002). No classical hydrogen bonds are observed in the crystal structure.

### Experimental

Title compound was published methods (Er *et al.*, 2009). Crystals were grown by slow evaporation of an dimethylformamide/alcohol mixed solution.

### Refinement

The hydrogen atoms were placed in calculated positions with C—H = 0.95–0.99 Å and refined using a riding model with fixed isotropic displacement parameters [*U*_iso_(H) = 1.5*U*_eq_(C) for the methyl groups and 1.2*U*_eq_(C) for the other H atoms]. The molecules are disordered about an inversion center, therefore, the O1—C12/C12—C13 and C13—C14/C14—O1 distances are average values. The SIMU and DELU constraint instructions in *SHELXL97* were used atom C13 in order to model the disorder properly during the refinement. For C13B the ISOR instruction was used as otherwise it went non-positive definite. The displacement parameters of the pairs C12/C12B and C14/C14B were set equal using the EADP instruction.

### Crystal data

**Table:**

C_50_H_60_Cl_8_O_12_	*Z* = 1
*M**_r_* = 1136.58	*F*(000) = 592
Triclinic, *P*1	*D*_x_ = 1.411 Mg m^−^^3^
Hall symbol: -P 1	Mo *K*α radiation, λ = 0.71073 Å
*a* = 8.0626 (3) Å	Cell parameters from 4477 reflections
*b* = 12.8693 (5) Å	θ = 3.0–29.4°
*c* = 13.9968 (6) Å	µ = 0.48 mm^−^^1^
α = 97.425 (3)°	*T* = 123 K
β = 102.878 (3)°	Plate, colorless
γ = 105.391 (3)°	0.62 × 0.19 × 0.07 mm
*V* = 1337.29 (9) Å^3^	

### Data collection

**Table:**

Agilent Xcalibur (Ruby, Gemini) diffractometer	6125 independent reflections
Radiation source: Enhance (Cu) X-ray Source	5231 reflections with *I* > 2σ(*I*)
Graphite monochromator	*R*_int_ = 0.020
Detector resolution: 10.5081 pixels mm^-1^	θ_max_ = 29.5°, θ_min_ = 3.1°
ω scans	*h* = −8→11
Absorption correction: multi-scan [*CrysAlis RED* (Agilent, 2011), based on expressions derived from Clark & Reid (1995)]	*k* = −17→17
*T*_min_ = 0.755, *T*_max_ = 0.967	*l* = −18→17
10254 measured reflections	

### Refinement

**Table:**

Refinement on *F*^2^	Primary atom site location: structure-invariant direct methods
Least-squares matrix: full	Secondary atom site location: difference Fourier map
*R*[*F*^2^ > 2σ(*F*^2^)] = 0.038	Hydrogen site location: inferred from neighbouring sites
*wR*(*F*^2^) = 0.090	H-atom parameters constrained
*S* = 1.03	*w* = 1/[σ^2^(*F*_o_^2^) + (0.0366*P*)^2^ + 0.6175*P*] where *P* = (*F*_o_^2^ + 2*F*_c_^2^)/3
6125 reflections	(Δ/σ)_max_ = 0.001
336 parameters	Δρ_max_ = 0.45 e Å^−^^3^
12 restraints	Δρ_min_ = −0.39 e Å^−^^3^

### Special details

**Table:**

Experimental. Absorption correction: CrysAlis RED, (Agilent, 2011) Empirical absorption correction using spherical harmonics, implemented in SCALE3 ABSPACK scaling algorithm. (Clark & Reid, 1995).
Geometry. All e.s.d.'s (except the e.s.d. in the dihedral angle between two l.s. planes) are estimated using the full covariance matrix. The cell e.s.d.'s are taken into account individually in the estimation of e.s.d.'s in distances, angles and torsion angles; correlations between e.s.d.'s in cell parameters are only used when they are defined by crystal symmetry. An approximate (isotropic) treatment of cell e.s.d.'s is used for estimating e.s.d.'s involving l.s. planes.
Refinement. Refinement of *F*^2^ against ALL reflections. The weighted *R*-factor *wR* and goodness of fit *S* are based on *F*^2^, conventional *R*-factors *R* are based on *F*, with *F* set to zero for negative *F*^2^. The threshold expression of *F*^2^ > σ(*F*^2^) is used only for calculating *R*-factors(gt) *etc*. and is not relevant to the choice of reflections for refinement. *R*-factors based on *F*^2^ are statistically about twice as large as those based on *F*, and *R*- factors based on ALL data will be even larger.

### Fractional atomic coordinates and isotropic or equivalent isotropic displacement parameters (Å^2^)

**Table:**

	*x*	*y*	*z*	*U*_iso_*/*U*_eq_	Occ. (<1)
Cl1	−0.23088 (6)	0.18327 (3)	−0.05330 (3)	0.03455 (11)	
Cl2	0.27679 (5)	0.55748 (3)	0.14470 (3)	0.03249 (10)	
Cl3	1.09088 (5)	0.67178 (3)	0.63733 (3)	0.02509 (9)	
Cl4	1.49291 (5)	0.83171 (4)	0.39813 (3)	0.03397 (10)	
O1	0.06285 (14)	0.10425 (9)	0.28297 (8)	0.0261 (3)	
O2	0.36555 (14)	0.17140 (9)	0.28761 (8)	0.0224 (2)	
O3	0.38227 (14)	0.41369 (8)	0.29369 (8)	0.0246 (2)	
O4	0.69832 (14)	0.58377 (9)	0.23343 (8)	0.0247 (2)	
O5	0.83793 (15)	0.75433 (10)	0.20043 (8)	0.0284 (3)	
O6	0.78680 (12)	0.65010 (8)	0.46395 (8)	0.0199 (2)	
C1	0.23412 (18)	0.36585 (12)	0.21470 (11)	0.0181 (3)	
C2	0.17432 (19)	0.41902 (12)	0.13926 (11)	0.0207 (3)	
C3	0.03219 (19)	0.36340 (13)	0.05666 (12)	0.0219 (3)	
H3A	−0.0064	0.4001	0.0053	0.026*	
C4	−0.05207 (19)	0.25336 (13)	0.05072 (11)	0.0212 (3)	
C5	0.00081 (19)	0.19851 (12)	0.12452 (11)	0.0204 (3)	
H5A	−0.0606	0.1231	0.1193	0.024*	
C6	0.14491 (18)	0.25450 (12)	0.20676 (11)	0.0177 (3)	
C7	0.20623 (19)	0.19703 (12)	0.29041 (11)	0.0203 (3)	
H7A	0.2288	0.2474	0.3558	0.024*	
C8	0.0878 (2)	0.05009 (16)	0.36587 (14)	0.0360 (4)	
H8A	0.1526	0.1048	0.4286	0.043*	
H8B	0.1581	−0.0008	0.3557	0.043*	
C9	−0.0953 (2)	−0.01277 (15)	0.37163 (14)	0.0366 (4)	
H9A	−0.0838	−0.0623	0.4186	0.055*	
H9B	−0.1664	−0.0559	0.3052	0.055*	
H9C	−0.1545	0.0391	0.3948	0.055*	
C10	0.3566 (2)	0.10171 (17)	0.19734 (14)	0.0384 (4)	
H10A	0.3398	0.1403	0.1408	0.046*	
H10B	0.2538	0.0342	0.1828	0.046*	
C11	0.5243 (3)	0.07216 (18)	0.20881 (18)	0.0505 (6)	
H11A	0.5235	0.0302	0.1450	0.076*	
H11B	0.5340	0.0274	0.2601	0.076*	
H11C	0.6263	0.1394	0.2289	0.076*	
C12	0.3929 (3)	0.5092 (2)	0.36286 (19)	0.0173 (5)	0.828 (3)
H12A	0.2743	0.5051	0.3738	0.021*	0.828 (3)
H12B	0.4360	0.5768	0.3374	0.021*	0.828 (3)
C13	0.5229 (2)	0.50930 (13)	0.45844 (14)	0.0173 (4)	0.828 (3)
C14	0.7108 (3)	0.5318 (2)	0.44948 (19)	0.0156 (5)	0.828 (3)
H14A	0.7817	0.5029	0.5008	0.019*	0.828 (3)
H14B	0.7109	0.4957	0.3826	0.019*	0.828 (3)
C12B	0.3501 (18)	0.5066 (13)	0.3681 (11)	0.0173 (5)	0.172 (3)
H12C	0.4165	0.5802	0.3601	0.021*	0.172 (3)
H12D	0.2215	0.5003	0.3518	0.021*	0.172 (3)
C13B	0.5857 (9)	0.5064 (6)	0.5228 (6)	0.0069 (12)	0.172 (3)
C14B	0.7384 (19)	0.5280 (13)	0.4666 (12)	0.0156 (5)	0.172 (3)
H14C	0.8434	0.5096	0.5031	0.019*	0.172 (3)
H14D	0.6946	0.4831	0.3979	0.019*	0.172 (3)
C15	0.95214 (18)	0.68454 (11)	0.44614 (11)	0.0173 (3)	
C16	1.10747 (19)	0.70221 (12)	0.52180 (11)	0.0184 (3)	
C17	1.27632 (19)	0.74633 (12)	0.50857 (12)	0.0212 (3)	
H17A	1.3817	0.7582	0.5607	0.025*	
C18	1.28421 (19)	0.77210 (12)	0.41639 (12)	0.0217 (3)	
C19	1.13317 (19)	0.75490 (13)	0.33920 (12)	0.0223 (3)	
H19A	1.1437	0.7729	0.2767	0.027*	
C20	0.96562 (19)	0.71107 (12)	0.35371 (11)	0.0193 (3)	
C21	0.7944 (2)	0.69472 (13)	0.27337 (12)	0.0225 (3)	
H21A	0.7165	0.7284	0.3052	0.027*	
C22	0.7855 (2)	0.52048 (15)	0.18194 (14)	0.0321 (4)	
H22A	0.8899	0.5121	0.2295	0.039*	
H22B	0.8272	0.5574	0.1300	0.039*	
C23	0.6502 (3)	0.40991 (17)	0.13475 (16)	0.0435 (5)	
H23A	0.7047	0.3638	0.0986	0.065*	
H23B	0.5478	0.4195	0.0880	0.065*	
H23C	0.6101	0.3744	0.1870	0.065*	
C24	0.6835 (2)	0.76496 (16)	0.13328 (14)	0.0350 (4)	
H24A	0.6044	0.7868	0.1714	0.042*	
H24B	0.6157	0.6938	0.0871	0.042*	
C25	0.7450 (3)	0.85117 (19)	0.07503 (15)	0.0456 (5)	
H25A	0.6409	0.8612	0.0300	0.068*	
H25B	0.8198	0.8276	0.0357	0.068*	
H25C	0.8145	0.9209	0.1214	0.068*	

### Atomic displacement parameters (Å^2^)

**Table:**

	*U*^11^	*U*^22^	*U*^33^	*U*^12^	*U*^13^	*U*^23^
Cl1	0.0297 (2)	0.0299 (2)	0.0292 (2)	0.00642 (17)	−0.01299 (17)	−0.00331 (17)
Cl2	0.02656 (19)	0.02333 (18)	0.0371 (2)	−0.00010 (16)	−0.00738 (17)	0.01357 (17)
Cl3	0.02481 (17)	0.03109 (19)	0.02040 (18)	0.00847 (15)	0.00794 (14)	0.00553 (15)
Cl4	0.01604 (16)	0.0430 (2)	0.0429 (2)	0.00276 (16)	0.01405 (16)	0.01116 (19)
O1	0.0259 (5)	0.0241 (5)	0.0267 (6)	0.0042 (5)	0.0045 (5)	0.0113 (5)
O2	0.0229 (5)	0.0233 (5)	0.0196 (5)	0.0105 (4)	0.0005 (4)	0.0010 (4)
O3	0.0228 (5)	0.0194 (5)	0.0229 (6)	0.0075 (4)	−0.0080 (4)	−0.0036 (4)
O4	0.0190 (5)	0.0270 (5)	0.0270 (6)	0.0078 (4)	0.0050 (4)	0.0022 (5)
O5	0.0246 (5)	0.0366 (6)	0.0276 (6)	0.0123 (5)	0.0057 (5)	0.0150 (5)
O6	0.0138 (4)	0.0155 (5)	0.0317 (6)	0.0032 (4)	0.0103 (4)	0.0041 (4)
C1	0.0154 (6)	0.0211 (7)	0.0152 (7)	0.0064 (6)	−0.0001 (5)	0.0000 (6)
C2	0.0178 (7)	0.0185 (7)	0.0231 (8)	0.0041 (6)	0.0016 (6)	0.0043 (6)
C3	0.0193 (7)	0.0258 (7)	0.0207 (7)	0.0094 (6)	0.0016 (6)	0.0064 (6)
C4	0.0170 (7)	0.0249 (7)	0.0169 (7)	0.0075 (6)	−0.0024 (6)	−0.0030 (6)
C5	0.0194 (7)	0.0183 (7)	0.0214 (7)	0.0058 (6)	0.0027 (6)	0.0012 (6)
C6	0.0173 (6)	0.0204 (7)	0.0170 (7)	0.0084 (6)	0.0053 (5)	0.0026 (6)
C7	0.0210 (7)	0.0197 (7)	0.0194 (7)	0.0064 (6)	0.0040 (6)	0.0030 (6)
C8	0.0341 (9)	0.0397 (9)	0.0376 (10)	0.0102 (8)	0.0088 (8)	0.0233 (8)
C9	0.0396 (10)	0.0340 (9)	0.0291 (9)	−0.0006 (8)	0.0074 (8)	0.0096 (8)
C10	0.0356 (9)	0.0479 (11)	0.0287 (9)	0.0231 (8)	−0.0003 (8)	−0.0092 (8)
C11	0.0298 (9)	0.0471 (12)	0.0633 (14)	0.0164 (9)	0.0021 (9)	−0.0197 (11)
C12	0.0173 (11)	0.0176 (7)	0.0158 (8)	0.0066 (8)	0.0015 (8)	0.0017 (6)
C13	0.0155 (8)	0.0130 (8)	0.0214 (10)	0.0031 (6)	0.0036 (7)	0.0016 (7)
C14	0.0124 (9)	0.0145 (7)	0.0211 (11)	0.0048 (7)	0.0049 (7)	0.0048 (7)
C12B	0.0173 (11)	0.0176 (7)	0.0158 (8)	0.0066 (8)	0.0015 (8)	0.0017 (6)
C13B	0.0069 (14)	0.0069 (14)	0.0065 (15)	0.0017 (9)	0.0020 (9)	0.0011 (9)
C14B	0.0124 (9)	0.0145 (7)	0.0211 (11)	0.0048 (7)	0.0049 (7)	0.0048 (7)
C15	0.0140 (6)	0.0137 (6)	0.0255 (7)	0.0041 (5)	0.0082 (6)	0.0033 (6)
C16	0.0190 (6)	0.0173 (7)	0.0199 (7)	0.0055 (5)	0.0077 (6)	0.0031 (6)
C17	0.0154 (6)	0.0215 (7)	0.0255 (8)	0.0061 (6)	0.0042 (6)	0.0017 (6)
C18	0.0137 (6)	0.0210 (7)	0.0314 (8)	0.0031 (6)	0.0107 (6)	0.0055 (6)
C19	0.0208 (7)	0.0258 (7)	0.0240 (8)	0.0080 (6)	0.0097 (6)	0.0095 (6)
C20	0.0170 (6)	0.0182 (7)	0.0234 (7)	0.0064 (6)	0.0053 (6)	0.0042 (6)
C21	0.0181 (7)	0.0258 (7)	0.0246 (8)	0.0070 (6)	0.0058 (6)	0.0082 (6)
C22	0.0277 (8)	0.0348 (9)	0.0354 (9)	0.0118 (7)	0.0120 (7)	0.0013 (8)
C23	0.0336 (9)	0.0421 (11)	0.0464 (12)	0.0112 (8)	0.0071 (9)	−0.0131 (9)
C24	0.0313 (9)	0.0422 (10)	0.0293 (9)	0.0134 (8)	−0.0009 (7)	0.0109 (8)
C25	0.0484 (11)	0.0707 (13)	0.0388 (10)	0.0367 (10)	0.0196 (9)	0.0309 (10)

### Geometric parameters (Å, º)

**Table:**

Cl1—C4	1.7454 (15)	C11—H11C	0.9800
Cl2—C2	1.7376 (15)	C12—C13	1.504 (3)
Cl3—C16	1.7374 (15)	C12—H12A	0.9900
Cl4—C18	1.7442 (14)	C12—H12B	0.9900
O1—C7	1.3980 (17)	C13—C13^i^	1.328 (4)
O1—C8	1.433 (2)	C13—C14	1.502 (3)
O2—C7	1.4158 (18)	C14—H14A	0.9900
O2—C10	1.428 (2)	C14—H14B	0.9900
O3—C1	1.3686 (17)	C12B—C13B^i^	1.546 (17)
O3—C12	1.434 (3)	C12B—H12C	0.9900
O3—C12B	1.595 (15)	C12B—H12D	0.9900
O4—C21	1.4018 (18)	C13B—C13B^i^	1.342 (15)
O4—C22	1.433 (2)	C13B—C12B^i^	1.546 (17)
O5—C21	1.4047 (19)	C13B—C14B	1.587 (18)
O5—C24	1.433 (2)	C14B—H14C	0.9900
O6—C15	1.3779 (16)	C14B—H14D	0.9900
O6—C14	1.451 (3)	C15—C16	1.392 (2)
O6—C14B	1.522 (16)	C15—C20	1.400 (2)
C1—C2	1.397 (2)	C16—C17	1.393 (2)
C1—C6	1.400 (2)	C17—C18	1.384 (2)
C2—C3	1.386 (2)	C17—H17A	0.9500
C3—C4	1.381 (2)	C18—C19	1.381 (2)
C3—H3A	0.9500	C19—C20	1.390 (2)
C4—C5	1.378 (2)	C19—H19A	0.9500
C5—C6	1.393 (2)	C20—C21	1.519 (2)
C5—H5A	0.9500	C21—H21A	1.0000
C6—C7	1.524 (2)	C22—C23	1.504 (3)
C7—H7A	1.0000	C22—H22A	0.9900
C8—C9	1.509 (2)	C22—H22B	0.9900
C8—H8A	0.9900	C23—H23A	0.9800
C8—H8B	0.9900	C23—H23B	0.9800
C9—H9A	0.9800	C23—H23C	0.9800
C9—H9B	0.9800	C24—C25	1.505 (3)
C9—H9C	0.9800	C24—H24A	0.9900
C10—C11	1.479 (3)	C24—H24B	0.9900
C10—H10A	0.9900	C25—H25A	0.9800
C10—H10B	0.9900	C25—H25B	0.9800
C11—H11A	0.9800	C25—H25C	0.9800
C11—H11B	0.9800		
			
C7—O1—C8	113.60 (12)	C13—C14—H14A	110.2
C7—O2—C10	115.03 (12)	O6—C14—H14B	110.2
C1—O3—C12	120.44 (13)	C13—C14—H14B	110.2
C1—O3—C12B	110.8 (5)	H14A—C14—H14B	108.5
C21—O4—C22	116.34 (12)	C13B^i^—C12B—O3	109.8 (10)
C21—O5—C24	112.49 (12)	C13B^i^—C12B—H12C	109.7
C15—O6—C14	114.90 (14)	O3—C12B—H12C	109.7
C15—O6—C14B	109.8 (6)	C13B^i^—C12B—H12D	109.7
O3—C1—C2	124.19 (13)	O3—C12B—H12D	109.7
O3—C1—C6	117.07 (13)	H12C—C12B—H12D	108.2
C2—C1—C6	118.61 (13)	C13B^i^—C13B—C12B^i^	123.3 (10)
C3—C2—C1	121.46 (14)	C13B^i^—C13B—C14B	122.6 (10)
C3—C2—Cl2	117.32 (12)	C12B^i^—C13B—C14B	113.9 (9)
C1—C2—Cl2	121.21 (11)	O6—C14B—C13B	105.5 (10)
C4—C3—C2	118.47 (14)	O6—C14B—H14C	110.6
C4—C3—H3A	120.8	C13B—C14B—H14C	110.6
C2—C3—H3A	120.8	O6—C14B—H14D	110.6
C5—C4—C3	121.80 (14)	C13B—C14B—H14D	110.6
C5—C4—Cl1	119.71 (12)	H14C—C14B—H14D	108.8
C3—C4—Cl1	118.50 (12)	O6—C15—C16	120.73 (13)
C4—C5—C6	119.47 (14)	O6—C15—C20	119.87 (13)
C4—C5—H5A	120.3	C16—C15—C20	119.18 (12)
C6—C5—H5A	120.3	C15—C16—C17	121.93 (14)
C5—C6—C1	120.17 (13)	C15—C16—Cl3	119.22 (11)
C5—C6—C7	121.03 (13)	C17—C16—Cl3	118.82 (11)
C1—C6—C7	118.79 (12)	C18—C17—C16	117.21 (14)
O1—C7—O2	112.96 (12)	C18—C17—H17A	121.4
O1—C7—C6	106.45 (11)	C16—C17—H17A	121.4
O2—C7—C6	113.03 (12)	C19—C18—C17	122.55 (13)
O1—C7—H7A	108.1	C19—C18—Cl4	118.80 (12)
O2—C7—H7A	108.1	C17—C18—Cl4	118.63 (11)
C6—C7—H7A	108.1	C18—C19—C20	119.52 (14)
O1—C8—C9	107.14 (14)	C18—C19—H19A	120.2
O1—C8—H8A	110.3	C20—C19—H19A	120.2
C9—C8—H8A	110.3	C19—C20—C15	119.61 (13)
O1—C8—H8B	110.3	C19—C20—C21	122.06 (13)
C9—C8—H8B	110.3	C15—C20—C21	118.30 (12)
H8A—C8—H8B	108.5	O4—C21—O5	113.06 (13)
C8—C9—H9A	109.5	O4—C21—C20	113.33 (12)
C8—C9—H9B	109.5	O5—C21—C20	108.04 (12)
H9A—C9—H9B	109.5	O4—C21—H21A	107.4
C8—C9—H9C	109.5	O5—C21—H21A	107.4
H9A—C9—H9C	109.5	C20—C21—H21A	107.4
H9B—C9—H9C	109.5	O4—C22—C23	106.83 (14)
O2—C10—C11	109.21 (15)	O4—C22—H22A	110.4
O2—C10—H10A	109.8	C23—C22—H22A	110.4
C11—C10—H10A	109.8	O4—C22—H22B	110.4
O2—C10—H10B	109.8	C23—C22—H22B	110.4
C11—C10—H10B	109.8	H22A—C22—H22B	108.6
H10A—C10—H10B	108.3	C22—C23—H23A	109.5
C10—C11—H11A	109.5	C22—C23—H23B	109.5
C10—C11—H11B	109.5	H23A—C23—H23B	109.5
H11A—C11—H11B	109.5	C22—C23—H23C	109.5
C10—C11—H11C	109.5	H23A—C23—H23C	109.5
H11A—C11—H11C	109.5	H23B—C23—H23C	109.5
H11B—C11—H11C	109.5	O5—C24—C25	108.28 (14)
O3—C12—C13	105.59 (18)	O5—C24—H24A	110.0
O3—C12—H12A	110.6	C25—C24—H24A	110.0
C13—C12—H12A	110.6	O5—C24—H24B	110.0
O3—C12—H12B	110.6	C25—C24—H24B	110.0
C13—C12—H12B	110.6	H24A—C24—H24B	108.4
H12A—C12—H12B	108.8	C24—C25—H25A	109.5
C13^i^—C13—C14	123.5 (2)	C24—C25—H25B	109.5
C13^i^—C13—C12	123.9 (2)	H25A—C25—H25B	109.5
C14—C13—C12	112.58 (17)	C24—C25—H25C	109.5
O6—C14—C13	107.35 (19)	H25A—C25—H25C	109.5
O6—C14—H14A	110.2	H25B—C25—H25C	109.5
			
C12—O3—C1—C2	60.8 (2)	C12—C13—C14—O6	82.5 (2)
C12B—O3—C1—C2	69.3 (6)	C1—O3—C12B—C13B^i^	133.7 (6)
C12—O3—C1—C6	−123.32 (17)	C12—O3—C12B—C13B^i^	−83 (3)
C12B—O3—C1—C6	−114.9 (6)	C15—O6—C14B—C13B	161.1 (6)
O3—C1—C2—C3	174.42 (14)	C14—O6—C14B—C13B	−80 (4)
C6—C1—C2—C3	−1.4 (2)	C13B^i^—C13B—C14B—O6	80.1 (12)
O3—C1—C2—Cl2	−5.0 (2)	C12B^i^—C13B—C14B—O6	−104.0 (10)
C6—C1—C2—Cl2	179.27 (11)	C14—O6—C15—C16	−87.63 (18)
C1—C2—C3—C4	0.9 (2)	C14B—O6—C15—C16	−76.5 (6)
Cl2—C2—C3—C4	−179.74 (12)	C14—O6—C15—C20	97.79 (17)
C2—C3—C4—C5	0.4 (2)	C14B—O6—C15—C20	108.9 (6)
C2—C3—C4—Cl1	−179.94 (12)	O6—C15—C16—C17	−174.14 (13)
C3—C4—C5—C6	−1.1 (2)	C20—C15—C16—C17	0.5 (2)
Cl1—C4—C5—C6	179.24 (12)	O6—C15—C16—Cl3	4.17 (19)
C4—C5—C6—C1	0.6 (2)	C20—C15—C16—Cl3	178.78 (11)
C4—C5—C6—C7	179.39 (14)	C15—C16—C17—C18	−0.1 (2)
O3—C1—C6—C5	−175.45 (13)	Cl3—C16—C17—C18	−178.42 (11)
C2—C1—C6—C5	0.6 (2)	C16—C17—C18—C19	−0.5 (2)
O3—C1—C6—C7	5.7 (2)	C16—C17—C18—Cl4	177.93 (11)
C2—C1—C6—C7	−178.22 (14)	C17—C18—C19—C20	0.6 (2)
C8—O1—C7—O2	64.73 (17)	Cl4—C18—C19—C20	−177.74 (12)
C8—O1—C7—C6	−170.66 (13)	C18—C19—C20—C15	−0.3 (2)
C10—O2—C7—O1	61.96 (17)	C18—C19—C20—C21	177.35 (14)
C10—O2—C7—C6	−58.98 (17)	O6—C15—C20—C19	174.37 (13)
C5—C6—C7—O1	−19.60 (19)	C16—C15—C20—C19	−0.3 (2)
C1—C6—C7—O1	159.23 (13)	O6—C15—C20—C21	−3.3 (2)
C5—C6—C7—O2	104.97 (15)	C16—C15—C20—C21	−177.98 (13)
C1—C6—C7—O2	−76.20 (17)	C22—O4—C21—O5	60.38 (17)
C7—O1—C8—C9	155.99 (14)	C22—O4—C21—C20	−63.00 (18)
C7—O2—C10—C11	−173.98 (16)	C24—O5—C21—O4	66.86 (17)
C1—O3—C12—C13	155.76 (13)	C24—O5—C21—C20	−166.88 (13)
C12B—O3—C12—C13	116 (3)	C19—C20—C21—O4	111.86 (16)
O3—C12—C13—C13^i^	−110.9 (2)	C15—C20—C21—O4	−70.51 (18)
O3—C12—C13—C14	68.6 (2)	C19—C20—C21—O5	−14.2 (2)
C15—O6—C14—C13	−172.50 (14)	C15—C20—C21—O5	163.40 (13)
C14B—O6—C14—C13	122 (4)	C21—O4—C22—C23	−172.83 (15)
C13^i^—C13—C14—O6	−98.1 (3)	C21—O5—C24—C25	166.58 (15)

Symmetry code: (i) −*x*+1, −*y*+1, −*z*+1.

### Hydrogen-bond geometry (Å, º)

**Table:**

*D*—H···*A*	*D*—H	H···*A*	*D*···*A*	*D*—H···*A*
C12—H12*B*···Cl2	0.99	2.66	3.180 (3)	113
C14—H14*B*···O3	0.99	2.53	2.918 (2)	103
C14—H14*B*···O4	0.99	2.50	3.166 (3)	125
C17—H17*A*···O2^ii^	0.95	2.47	3.3943 (18)	166

Symmetry code: (ii) −*x*+2, −*y*+1, −*z*+1.
